# A Case Report: Effects of a ketogenic diet on PTEN mutation-associated autism spectrum disorder

**DOI:** 10.3389/fnut.2026.1721018

**Published:** 2026-01-21

**Authors:** Yu Wang, Yanshuo Guo, Mengna Zhu, Linlin Zhang, Lihong Wang, Zhiyue Liu, Yongheng Zhao, Zihan Yan, Jiayi Gao, Aihua Cao

**Affiliations:** 1 Department of Pediatrics, Qilu Hospital of Shandong University, Jinan, Shandong, China; 2 Department of Pediatrics, Qilu Hospital of Shandong University Dezhou Hospital, Dezhou, Shandong, China

**Keywords:** autism spectrum disorder, child, ketogenic diet, PTEN, repetitive transcranial magneticstimulation

## Abstract

**Background:**

Autism spectrum disorder (ASD), a neurodevelopmental condition strongly associated with PTEN mutations, demonstrates limited responsiveness to current therapeutic approaches. Ketogenic diet (KD) has emerged as a promising dietary intervention in neurological disorders, including epilepsy and ASD.

**Objective:**

This case report investigates the clinical efficacy and potential mechanisms of KD in an ASD patient with a PTEN mutation, providing evidence for genetics-guided precision nutrition therapy.

**Methods:**

We present a 7-year, 1-month-old male with ASD and a pathogenic heterozygous PTEN p. Arg130Gln mutation, who showed suboptimal response to conventional treatments. He received a modified Atkins diet (60% fat, 30% protein, 10% carbohydrates) for 1 month, followed by 5 months of combined KD and repetitive transcranial magnetic stimulation (rTMS). Efficacy was evaluated through serial behavioral assessments, metabolic markers (blood ketones, inflammatory cytokines), and EEG monitoring. Safety parameters were documented.

**Results:**

During the intervention, mean blood glucose was 4.4 mmol/L and ketones averaged 2.4 mmol/L. Significant improvements in behavior, sleep, and metabolic profiles were observed. Parents noted symptomatic improvement by day 6. Quantitative assessments (behavioral scales, metabolic markers, EEG) confirmed substantial progress after 1 month of KD and 5 months of KD-rTMS versus baseline. While the rate of improvement diminished during combined therapy compared to initial KD monotherapy, excitatory behaviors (e.g., screaming, uncontrolled running, sleep-onset difficulties) were markedly reduced compared to prior rTMS-only treatment. No adverse events (e.g., hypoglycemia, ketoacidosis) occurred, and the regimen was well-tolerated.

**Conclusion:**

This study provides preliminary clinical evidence for KD in PTEN-related ASD, suggesting potential modulation of the PI3K/AKT/mTOR pathway and neuroinflammation. Although limited by its single-case design and short duration, it offers a novel therapeutic approach for PTEN-mutant ASD. Multicenter randomized controlled trials are warranted to validate efficacy and safety.

## 1 Introduction

Autism Spectrum Disorder (ASD) is a neurodevelopmental disorder that manifests in early childhood, with core characteristics including social interaction and communication impairments, restricted interests, and repetitive and restricted behaviors (1). The prevalence of ASD is increasing year by year (2). However, the etiology of ASD remains incompletely understood. It is widely believed that both genetic and environmental factors contribute to its development, making ASD a complex multifactorial disease (3). Notably, genetic factors play a significant role in the pathogenesis of ASD, as evidenced by family and twin studies of ASD demonstrating a high genetic risk (4–6). Of the numerous genetic variants associated with ASD, mutations in the phosphatase and tensin homolog (PTEN) gene represent a particularly compelling etiological factor requiring further investigation.

PTEN, located on chromosome 10 (10q23.3), is a well-known tumor suppressor gene that regulates multiple cellular processes frequently dysregulated in cancer (7). Diseases associated with germline PTEN variants are collectively referred to as PTEN hamartoma tumor syndrome (PHTS), which encompasses Cowden syndrome (CS), Bannayan-Riley-Ruvalcaba syndrome (BRRS), PTEN-related Proteus syndrome (PS), and PTEN-related Proteus-like syndrome. Studies have shown that this variation is associated with an increased lifetime risk of PHTS component cancers such as breast, thyroid, endometrial, kidney, and colon cancers, as well as melanoma (8). In addition, since ASD and PTEN were initially linked in 2005 by Butler et al. (9) who reported that three of a group of eighteen individuals with ASD and macrocephaly had germline PTEN mutations, subsequent murine models and clinical cohorts have confirmed that PTEN is a recognized risk gene for ASD and neurodevelopmental disorders (10–12). A meta-analysis suggests that the prevalence of ASD or its characteristics in PTEN mutations population is approximately 25% (13). Although this value needs to consider the influence of publication bias and methodological heterogeneity, it still markedly exceeds the prevalence of ASD in the general population even under conservative assumptions (14, 15).

While the pathogenic role of PTEN mutations in ASD development is well-documented, current clinical interventions primarily rely on behavioral modification and rehabilitation therapies. The substantial disease burden during management and the suboptimal therapeutic outcomes necessitate the urgent exploration of more efficient treatment strategies. The ketogenic diet (KD) – a nutritional regimen characterized by severe carbohydrate restriction, high fat intake, and controlled protein provision - has garnered significant attention in recent years. Under KD, the body utilizes ketones (acetoacetic acid, beta-hydroxybutyric acid, and acetone) as the primary energy source instead of carbohydrates (16). Originally conceptualized by Dr. Russell Wilder in 1921 for pediatric epilepsy management (17), over the past century, the clinical applications of KD have expanded beyond refractory epilepsy to encompass various neurological diseases, including ASD (17–19). However, its therapeutic potential specifically in ASD patients with PTEN mutations remains to be systematically investigated.

We present a case of a child with ASD harboring a PTEN gene mutation. Following suboptimal therapeutic response to conventional therapies, the patient demonstrated marked improvements in social communication, verbal expression, and stereotypic behaviors after KD intervention. This case provides preliminary evidence supporting the potential therapeutic application of KD in PTEN mutation-associated ASD. Integrated analysis of genotype–phenotype correlations and metabolic regulatory mechanisms leads us to hypothesize that KD may ameliorate neurodevelopmental impairments by modulating aberrant PTEN/mTOR pathway activity, thereby offering novel therapeutic avenues for genetically distinct ASD subtypes and advancing theoretical frameworks in precision nutritional medicine.

## 2 Case presentations

### 2.1 Pre-KD

**Figure 1:**
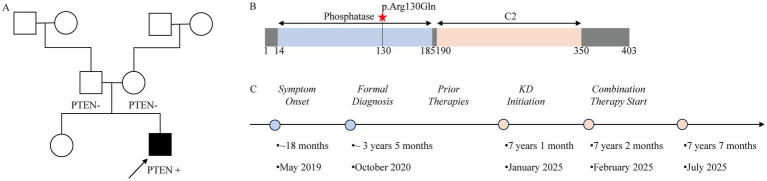
Genetic and clinical profile of the proband with a *de novo* PTEN p.Arg130Gln mutation. **(A)** Three-generation pedigree. The arrow indicates the male proband. The filled symbol denotes the proband carrying the heterozygous pathogenic PTEN variant (c.389G > A, p.Arg130Gln) and presenting with ASD and macrocephaly. Unaffected family members are shown as open symbols. Genetic testing confirmed both parents did not carry the variant (“PTEN-”), supporting a de novo origin. **(B)** Schematic of the PTEN protein domain structure. The phosphatase core domain (blue) and C2 domain (light orange) are shown. The pathogenic p.Arg130Gln missense mutation is indicated by a red asterisk at residue 130 within the phosphatase domain. **(C)** Clinical timeline of disease course and interventions. Key assessment timepoints (light orange dots) include: Pre-KD, 1-Month KD, and 5-Month KD-rTMS.

A 7-year-1-month-old male patient was born via full-term cesarean delivery with a birth weight of 3.2 kg to a 54-year-old father and 50-year-old mother, showing uncomplicated perinatal course. He has a healthy 25-year-old elder sister, with no family history of neurobehavioral developmental disorders (Figure 1A). At approximately 18 months of age (May 2019), caregivers noted marked language delay (limited to occasional vocalizations of “baba” and “mama” without intentional speech) and impaired social reciprocity (minimal eye contact, non-response to name, exclusive use of hand-pulling for requests, and absence of peer interactions). October 2019 evaluations revealed: Gesell Developmental Schedules showing adaptive DQ62, gross motor DQ69, fine motor DQ72, language DQ60, and personal-social DQ57; M-CHAT autism screening scoring 15; non-contrast cranial MRI demonstrating bilateral centrum semiovale and posterior periventricular hyperintense T2 signals suggestive of perivascular spaces, with localized ventricular enlargement.

Initial management only involved home-based early intervention until October 2020, when macrocephaly (head circumference 55 cm) and stereotypic behaviors emerged, including fascination with light stimuli, wheel-rotation watching, door/light switching, hand-flapping, and hand-gazing. The patient was formally diagnosed with ASD, with genetic testing identifying a PTEN mutation (Table 1; Figure 1B). Subsequent multi-modal therapy comprised Behavioral-Structured-Relational intervention, professional rehabilitation, repetitive transcranial magnetic stimulation (rTMS), and pharmacotherapy (citicoline, idebenone, aripiprazole, etc.). It is noteworthy that during rTMS monotherapy, pronounced agitation (e.g., screaming, non-purposeful running, and sleep initiation difficulties) was observed peri-stimulation, yet these adverse effects subsided during treatment-free intervals, where concurrent clinical improvements were observed. By January 2025 (prior to KD initiation), cognitive, communicative, and social functioning remained significantly delayed relative to peers, characterized by minimal spontaneous speech, persistent stereotypic behaviors, chronic sleep disturbances (nocturnal awakenings), and constipation. Driven by the identified PTEN mutation and classic ASD phenotype, the KD was pursued as a therapeutic intervention. The patient’s clinical history and the sequence of interventions are summarized in Figure 1C.

**Table 1:** Genetic characterization of the PTEN variant in the ASD patient.

Gene	Chromosomal location	Position	Reference transcript	cDNA level	Protein level	Status	Variant classification	Father and mother
PTEN	chr10:89692905	Exon5	NM_000314.6	c.389G>A	p. (Arg130Gln)	heterozygous	Pathogenic	Not detected

### 2.2 Treatment protocol

KD Protocol: The present case implemented a modified Atkins diet (MAD) protocol with caloric distribution at 60% fat, 30% protein, and 10% carbohydrates. Pre-intervention systematic caregiver education encompassed MAD mechanisms, adverse event management (e.g., hypoglycemia, ketosis), home dietary protocols, with emphasis on carbohydrate quantification techniques, caloric requirement estimation, protein sufficiency, and micronutrient supplementation.

A dynamic adjustment protocol was employed: During the initial phase (days 1–3), thrice-daily glucose (target 3.9–5 mmol/L) and ketone (target 2–5 mmol/L) monitoring was performed under fasting/postprandial (2 h) conditions, with nursing specialists demonstrating and training caregivers in point-of-care testing. Upon achieving metabolic stability, monitoring was sequentially de-escalated to daily →three times per week→ weekly measurements, complemented by weekly clinical evaluations where dietitians refined dietary plans based on anthropometric trends and ketotic status. During intervention, metabolic parameters stabilized within target ranges (mean glucose 4.4 mmol/L, ketones 2.4 mmol/L), with no documented hypoglycemia, ketoacidosis, emesis, or diarrhea, demonstrating favorable tolerability. All safety parameters (Complete Blood Count, hepatic/renal function, cardiac enzymes, lipid profile, bio-chemistry) remained within normal ranges throughout the intervention.

rTMS Protocol: rTMS was administered using continuous theta-burst stimulation (cTBS). Stimulation was targeted at the primary motor cortex (M1). The cTBS protocol consisted of bursts of 3 pulses at 50 Hz, repeated at 5 Hz. Each treatment session delivered a total of 1800 pulses over 40 s without interruption. This protocol was identical to that used during the rTMS monotherapy phase prior to KD. During the 5-month combined intervention phase, a treatment cycle comprised 10 such sessions per day, with an inter-session interval of 50 min, delivered over 5 consecutive days. This intensive cycle was repeated once per month. Throughout this period, the patient maintained the established KD protocol.

### 2.3 1-Month KD monotherapy outcomes

**Figure 2:**
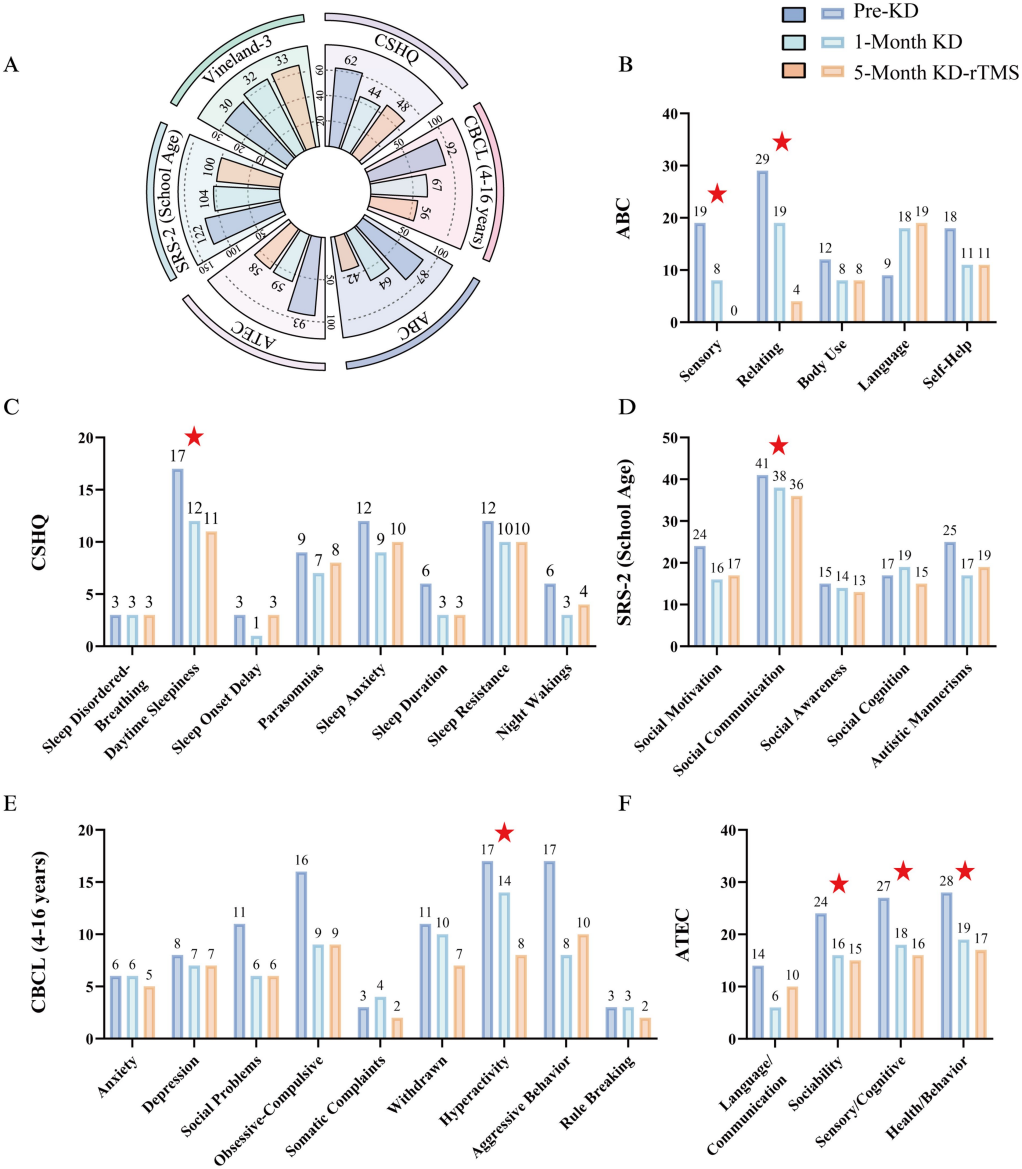
Comprehensive longitudinal assessment of clinical outcomes: Pre-KD, 1-month KD monotherapy, and 5-month combined KD-rTMS intervention. **(A)** Total scores comparison: Shows differences in total scores of Children’s Sleep Habits Questionnaire (CSHQ), Autism Behavior Checklist (ABC), Child Behavior Checklist (CBCL) (4–16 years), Autism Treatment Evaluation Checklist (ATEC), Social Responsiveness Scale (SRS-2) (School Age), and Vineland Adaptive Behavior Scales (Vineland-3) during the three assessment phases. **(B–F)** Subscale comparisons: Present score differences across subscales of the ABC, CSHQ, SRS-2 (School age), CBCL (4–16 years) and ATEC during the three treatment periods. Red asterisks highlight the subscales within each assessment tool that showed the most pronounced improvement.

Following a 1-month KD intervention, the patient exhibited progressive multi-systemic improvements. By day 6 of intervention, improved emotional stability and reduced stereotypies (e.g., vocal tics) were observed, accompanied by initial sleep consolidation manifesting as decreased nocturnal awakenings. By day 11, sleep latency post-awakening shortened significantly, with enhanced attentional capacity enabling completion of structured learning tasks. At day 15, marked progress in fine motor skills emerged, evidenced by independent pine nut shelling and 50 consecutive tracing exercises, alongside consolidated sleep architecture without nocturnal disturbances. By intervention endpoint (day 29), novel gross motor skill acquisition (continuous rope-jumping, scooter braking) and sustained focus during puzzle/block assembly tasks were documented. Concomitant improvements included: expanded verbal out-put, enhanced social reciprocity (eye contact, name response), cognitive command-following, and stool consistency normalization (Bristol scale 2/3 → 3/4). Parental satisfaction with global functioning was corroborated by standardized scale improvements (Figure 2), substantiating KD’s therapeutic efficacy.

Beyond caregiver-reported outcomes, further laboratory tests showed that the KD significantly improved the metabolic status and neurological function of the children. Free fatty acids (FFA) increased from 34.1 to 1123.6 μmoL/dL, with β-hydroxybutyrate (BHB) surging from 0.07 to 2.27 mmol/L, confirming robust ketosis establishment. Concurrently, inflammatory markers decreased: lactate dehydrogenase (LDH) 323 → 293 U/L; interleukin-2 receptor (IL-2R) 720 → 639 U/mL; TNF-*α* 10.6 → 8.72 pg./mL, demonstrating KD’s anti-inflammatory properties. Notably, electroencephalographic normalization (from borderline to typical patterns) evidenced KD-mediated neurofunctional regulation.

### 2.4 5-Month combined KD-rTMS efficacy

Given that prior rTMS monotherapy, despite transient adverse effects, was associated with observable clinical improvement, and considering the significant alleviation of core ASD symptoms following initial KD intervention, clinicians recommended a combined KD and rTMS. This approach hoped to achieve synergistic therapeutic effects while reducing associated adverse reactions. The rehabilitation regimen was rigorously maintained at identical intensity and frequency during: (i) the 6-month pre-KD baseline period, (ii) KD monotherapy phase, and (iii) subsequent 5-month combined KD-rTMS intervention. During the 5-month combined therapy, sustained improvements in core behavioral symptoms and peripheral inflammatory biomarkers were observed. Following the combined therapy, clinical assessment outcomes are detailed in Figure 1, with laboratory results indicating: LDH 250 U/L, IL-2R 503 U/mL, and TNF-*α* 9.47 pg./mL, electroencephalogram is normal. Notably, although progressive symptomatic alleviation was observed, the pace of improvement was less pronounced compared to the initial KD monotherapy phase (first treatment month). Strikingly, compared to the rTMS monotherapy phase prior to the KD, the patient demonstrated excellent tolerance during the combined KD-rTMS intervention period, with a significant reduction in excitatory adverse reactions. This observation suggests that the KD may modulate neuronal excitability to counteract the overstimulation effects potentially induced by rTMS. These findings provide preliminary clinical evidence for therapeutic strategies targeting PTEN mutation-associated ASD, warranting further investigation into their synergistic mechanisms in future studies.

## 3 Discussion

This case study demonstrates that KD intervention yielded significant therapeutic effects in an ASD patient harboring a PTEN gene mutation. The patient demonstrated marked improvements in core symptoms (e.g., stereotyped behaviors and social communication deficits), motor functions (particularly fine motor skills), and physiological parameters (including sleep quality and inflammatory responses). Notably, the combined KD and rTMS therapy resulted in a significant reduction in hyperexcitable behaviors compared to rTMS monotherapy. These findings provide substantial clinical evidence supporting precision nutritional therapy for PTEN mutation-associated ASD.

PTEN mutations are associated with a broad clinical spectrum, encompassing the classic features of PHTS and extending to reported phenotypes such as ASD with macrocephaly, intellectual disability, language delay, and digital anomalies (9, 20–24). The phenotype of our case aligns with these characteristic PTEN-related neurodevelopmental features. Notably, to our knowledge, this represents the first report of a structured KD intervention trial targeting core behavioral symptoms in a pediatric patient carrying the pathogenic PTEN p. Arg130Gln variant. Mechanistically, the p.Arg130Gln variant is located within the phosphatase domain of PTEN, which impairs its phosphatase activity (25, 26). Based on this mechanistic link, we propose a potential therapeutic mechanism whereby the KD may exert its effects by modulating the dysregulated PI3K/AKT/mTOR pathway. Previous studies have established that aberrant activation of the PI3K/AKT/mTOR pathway is strongly associated with ASD pathogenesis (27, 28), with PTEN functioning as a critical regulator of this pathway. PTEN mutations induce mTOR hyperactivation (29, 30), while KD-generated BHB attenuates this pathological activation through multiple mechanisms, thereby ameliorating associated physiological and behavioral abnormalities. Mechanistically, BHB mediates *β*-hydroxybutyrylation modification at lysine 108 of aldehyde dehydrogenase 2 (ALDH2), effectively suppressing mTOR signaling activity (31). Notably, hyperactivation of the mTOR pathway is closely linked to neuroinflammation (32, 33). In the present case, the observed reduction in the patient’s peripheral inflammatory cytokine levels aligns with the theoretical expectation that BHB, via the aforementioned mechanism, inhibits mTOR signaling and subsequently mitigates inflammation. Beyond the canonical PI3K/AKT/mTOR pathway, PTEN is also involved in regulating key neurodevelopmental processes such as synaptic plasticity and the development and function of GABAergic inhibitory neurons—processes that are closely linked to the pathophysiology of ASD (34–36). The KD may exert beneficial effects on these PTEN-related neural functions through multiple mechanisms. Supporting this, rodent studies further corroborate KD’s capacity to modulate the excitatory-inhibitory balance within neural circuits (37, 38). *Caenorhabditis elegans* neuromuscular models reveal that daf-18/PTEN orthologue mutations predominantly impair GABAergic neurotransmission development and function, while preserving cholinergic signaling integrity. BHB-enriched diets activate the DAF-16/FOXO signaling cascade, rescuing GABAergic deficits in daf-18/PTEN mutants (39). These mechanistic insights establish a biological foundation for addressing ASD core symptoms and comorbidities, reinforcing KD’s therapeutic potential for PTEN mutation- associated ASD.

Our findings contribute to the evolving landscape of precision nutrition in neurogenetics. A recent case report demonstrated the efficacy of KD in managing intractable seizures in a patient with developmental and epileptic encephalopathy caused by BAF53B mutations, underscoring KD’s utility in genetic epilepsy syndrome (40). Our case extends this paradigm by applying KD primarily for core behavioral symptoms of ASD in a patient with a PTEN mutation, suggesting a broader therapeutic scope. Furthermore, our choice of the MAD aligns with the precision nutrition framework, which advocates tailoring the KD variant to the patient’s age, clinical profile, and tolerability to optimize outcomes (41). While this study offers valuable clinical insights, several important limitations must be emphasized. First, it is crucial to state that KD is considered a symptom-management or compromise therapy, not a cure, for neurodevelopmental disorders. The single-case design severely limits generalizability of the results and cannot exclude confounding effects from individual variability. Furthermore, the co-intervention with rTMS complicates the interpretation of which therapy (or their combination) contributed to the observed improvements. Consequently, a fundamental question raised by this design—namely, whether the patient might have achieved comparable or even greater improvement from an extended period of KD monotherapy alone—cannot be answered. The open-label design introduces potential observer bias. Most importantly, the absence of molecular biomarker monitoring (e.g., mTOR pathway activity) restricts a direct mechanistic interpretation of KD’s actions in this patient.

Future investigations should employ multicenter randomized controlled trials to validate KD’s efficacy and safety profiles in PTEN-mutated ASD populations. Integration with metabolomic profiling could identify predictive biomarkers for treatment optimization and personalized nutritional strategies. Combinatorial approaches merging KD with mTOR inhibitors or neuromodulation techniques may expand therapeutic options for PTEN-related ASD. Specifically, well-controlled studies are warranted to directly compare the efficacy of KD monotherapy versus combined KD-rTMS intervention, in order to determine whether the combination offers additive or synergistic benefits for this population. This research illuminates novel treatment paradigms for PTEN-related ASD, particularly advocating KD as a viable option for treatment-resistant cases.

## 4 Conclusion

This case provides novel clinical evidence supporting KD as a potential targeted nutritional intervention for children with PTEN mutation-associated ASD. Following KD intervention under conditions of limited efficacy of conventional therapies, caregivers reported significant multidimensional improvements in the patient. While acknowledging certain limitations inherent to the study design, these findings establish preliminary rationale for KD’s therapeutic potential in PTEN-related neurodevelopmental disorders. Future investigations should employ rigorously controlled clinical trials and mechanistic studies to quantify KD’s impact on PTEN-mutated ASD symptomatology and elucidate its underlying mechanisms.

## Data Availability

The original contributions presented in the study are included in the article/supplementary material, further inquiries can be directed to the corresponding author/s.
